# Analysis of Lung Cancer Incidence in Non-Hispanic Black and White Americans using a Multistage Carcinogenesis Model

**DOI:** 10.1007/s10552-024-01936-7

**Published:** 2024-11-19

**Authors:** Sarah Skolnick, Pianpian Cao, Jihyoun Jeon, S. Lani Park, Daniel O. Stram, Loïc Le Marchand, Rafael Meza

**Affiliations:** 1 Department of Epidemiology, University of Michigan, Ann Arbor, MI USA; 2 Health Services Research, The University of Texas MD Anderson Cancer Center, Houston, TX USA; 3 Epidemiology Program, University of Hawaii Cancer Center, Honolulu, HI USA; 4 University of Southern California, Los Angeles, CA USA; 5 Integrative Oncology Program, BC Cancer Research Institute, 675 West 10th Avenue, Vancouver, BC Canada; 6 School of Population and Public Health, University of British Columbia, Vancouver, BC Canada

**Keywords:** Lung cancer, Disparities, Black/African Americans, Simulation model

## Abstract

**Purpose:**

There are complex and paradoxical patterns in lung cancer incidence by race/ethnicity and gender; compared to non-Hispanic White (NHW) males, non-Hispanic Black (NHB) males smoke fewer cigarettes per day and less frequently but have higher lung cancer rates. Similarly, NHB females are less likely to smoke but have comparable lung cancer rates to NHW females. We use a multistage carcinogenesis model to study the impact of smoking on lung cancer incidence in NHB and NHW individuals in the Multiethnic Cohort Study (MEC).

**Methods:**

The effects of smoking on the rates of lung tumor initiation, promotion, and malignant conversion, and the incidence of lung cancer in NHB versus NHW adults in the MEC were analyzed using the Two-Stage Clonal Expansion (TSCE) model. Maximum likelihood methods were used to estimate model parameters and assess differences by race/ethnicity, gender, and smoking history.

**Results:**

Smoking increased promotion and malignant conversion but did not affect tumor initiation. Non-smoking-related initiation, promotion, and malignant conversion and smoking-related promotion and malignant conversion differed by race/ethnicity and gender. Non-smoking-related initiation and malignant conversion were higher in NHB than NHW individuals, whereas promotion was lower in NHB individuals.

**Conclusion:**

Findings suggest that while smoking plays an important role in lung cancer risk, background risk not dependent on smoking also plays a significant and under-recognized role in explaining race/ethnicity differences. Ultimately, the resulting TSCE model will inform race/ethnicity-specific lung cancer natural history models to assess the impact of preventive interventions on US lung cancer outcomes and disparities by race/ethnicity.

**Supplementary Information:**

The online version contains supplementary material available at 10.1007/s10552-024-01936-7.

## Introduction

In 2024, an estimated 125,070 individuals will die of lung cancer, with 70–90% of such deaths attributed to smoking [1, 2]. There are complex and paradoxical patterns in lung cancer incidence by race/ethnicity and gender; compared to non-Hispanic White (NHW) males, non-Hispanic Black (NHB) males smoke fewer cigarettes per day and less frequently but have higher lung cancer rates [3–6]. Similarly, NHB females are less likely to smoke but have comparable rates of lung cancer to NHW females. Lung cancer risk is also known to depend on age, smoking duration, time since quit for former smokers, and timing of exposure [7–9]. To investigate these complex and time-dependent patterns in lung cancer incidence, we fitted a multistage carcinogenesis model, the Two-stage Clonal Expansion (TSCE) model, to the Multiethnic Cohort Study (MEC) data.

The TSCE model incorporates the impact of age-dependent carcinogen exposures, such as smoking, on the dynamics of lung cancer initiation, promotion, and malignant conversion, which allows for explicit examination and group comparisons of smoking-related and non-smoking-related (background) lung cancer risk. The TSCE model has been previously used to explore the relationship between smoking and lung cancer incidence and mortality in several prospective cohorts [7, 10–14]. These studies have suggested that smoking-related promotion is the primary mechanism of lung carcinogenesis. This study expands on previous work by exploring racial and ethnic differences in the age and time dynamics of smoking-related and background lung cancer incidence.

The MEC is an ideal data set to understand the differences in lung cancer incidence by race/ethnicity. The MEC is an ongoing study started in 1993 of 215,000 individuals recruited to investigate exposures related to chronic illnesses and cancer in an ethnically diverse cohort. Study questionnaires have collected detailed smoking information, allowing for the creation of individual smoking histories: cigarettes smoked per day, when the individual initiated, if and when the individual quit or relapsed. Additionally, the MEC is linked to Surveillance, Epidemiology, and End Results (SEER) data to obtain thorough and complete cancer incidence and mortality data.

We used likelihood-based methods to fit the TSCE model to lung cancer incidence of NHB and NHW individuals in MEC, allowing for differences in model parameters by race/ethnicity and gender. Using the final model, we investigated the impact of race/ethnicity on background and smoking-related lung cancer risk. Furthermore, we examined the effect of cigarettes per day, duration, and cessation on lung cancer risk by race/ethnicity and predicted incidence based on individual smoking history and race/ethnicity.

## Methods

### The Multiethnic Cohort Study (MEC)

The MEC is a National Cancer Institute (NCI)-funded longitudinal cohort created to investigate the development of cancer and other chronic illnesses [15, 16]. From 1993 to 1996, 215,000 individuals from Hawaii and Los Angeles were recruited. Individuals aged 45 to 75 at enrollment are being prospectively followed until death. Every five years, individuals are sent questionnaires that ask varying questions about risk factors [16]. The MEC is uniquely suited for this analysis due to its inclusion of large samples of individuals from racial and ethnic minorities, who are traditionally underrepresented in research studies, particularly NHB individuals. The MEC contains large samples of Black, White, Japanese American, Native Hawaiian, and Latino individuals. For this analysis, we focused on Black and White individuals.

We excluded individuals from analysis due to missing demographic data [16] or if they were missing information needed to determine their smoking history: age at initiation, number of cigarettes smoked per day, and age of quitting (if they were a former smoker). Additional information on exclusions is found in Supplementary Fig. 1. The final cohort used in this analysis consists of 81,586 individuals: 33,067 NHB and 48,519 NHW individuals.

### Smoking Histories

**Fig. 1:**
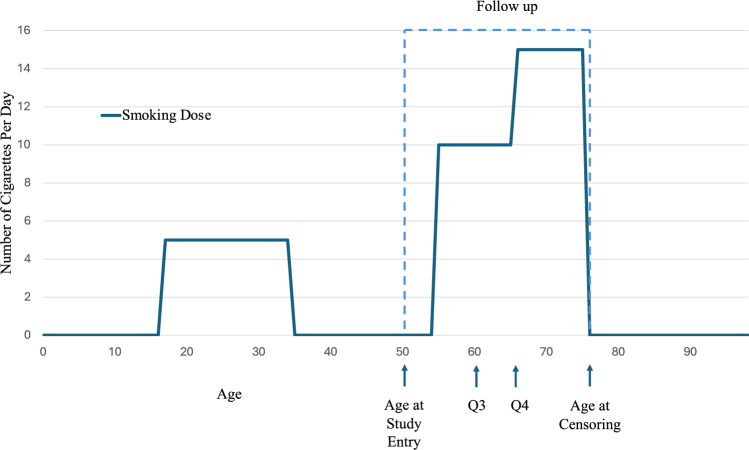
Example of the smoking history of a participant in the Multi-Ethnic Cohort (MEC)

We used smoking data from MEC questionnaires one (Q1, 1993–1996), three (Q3, 2003–2008), and four (Q4, 2008–2012) to reconstruct participants’ smoking histories. These questionnaires capture smoking history before individuals were enrolled, as well as ongoing changes in smoking for approximately two decades of follow-up (1993–2012). Q1 and Q3 asked respondents about age at initiation, number of cigarettes smoked per day, and age of quitting (if applicable), while Q4 collected information on whether an individual was a current smoker and the number of cigarettes the individual smoked. Figure 1 shows an example of an individual’s constructed smoking history. From this smoking history, yearly smoking dose information was incorporated into the TSCE model. Some variables necessary to construct a smoking history (i.e., number of cigarettes per day) were formatted categorically; for example, in Q1, individuals were asked if they had smoked less than or equal to 6, 6–10, 11–20, 21–30, and greater than or equal to 31 cigarettes per day. To incorporate these values into the model, we took the averages of these intervals to create a continuous variable.

Incomplete questionnaires and changes in behavior after the last completed questionnaire could lead to mismeasurement of smoking status. Similar to other tobacco health studies, which have followed participants for approximately 10 to 25 years after smoking assessments [9, 17, 18], in the main analysis, we censored individuals 10 years after their last smoking assessment. A table of the proportion of NHB and NHW with 1 and 2 follow-up questionnaires is provided in Supplementary Table 1.

**Table 1:** Characteristics of Multi-Ethnic Cohort (MEC) final sample

	Non-Hispanic Black	Non-Hispanic White	P
Lung cancer			
Not a case	31,908 (96.5%)	47,081 (97.0%)	5.268e-11
Case	1159 (3.5%)	1438 (3.0%)	
Sex			
Male	11,970 (36.2%)	22,384 (46.1%)	< 2.2e-16
Female	21,097 (63.8%)	26,135 (53.9%)	
Age at study entry			
Mean (SD)	61.8 (9.08)	59.5 (9.08)	< 2.2e-16
Median [min, max]	62.8 [45.0, 88.9]	59.2 [45.0, 87.6]	
Smoking status			
Never	12,891 (39.0%)	19,018 (39.2%)	< 2.2e-16
Former	12,553 (38.0%)	21,236 (43.8%)	
Current	7511 (22.7%)	8092 (16.7%)	
Missing	112 (0.3%)	173 (0.4%)	
Follow-up (years)			
Mean (SD)	14.2 (7.47)	16.7 (7.19)	< 2.2e-16
Median [min, max]	10.0 [0, 24.7]	19.6 [0, 24.7]	
Pack years at entry (pack-years)			
Mean (SD)	9.76 (13.3)	13.5 (17.5)	< 2.2e-16
Median [min, max]	3.88 [0, 63.6]	3.88 [0, 63.6]	
Missing	301 (0.9%)	372 (0.8%)	

The MEC is linked to the Hawaii and California SEER registries to obtain lung cancer case data. Deaths are obtained by linking to state death certificate files and the National Death Index [3, 16]. Smoking data were obtained up to Q4 which occurred approximately 12 to 19 years after baseline or Q1.

### Two-Stage Clonal Expansion (TSCE) Model

Multistage carcinogenesis models have been widely used to gain a better understanding of the underlying mechanisms of cancer development [7, 14, 19–26]. The TSCE model describes the development of carcinogenic cells following an initiation-promotion-progression paradigm [27]. Healthy cells become pre-cancerous or initiated, captured by the initiation rate. Pre-cancerous or initiated cells can die or multiply, captured by the promotion rate (cell division minus cell death rate). Finally, pre-cancerous cells can become cancerous, captured by the progression (malignant conversion) rate. The time dimension for the rates presented in this paper is age in years.

Yearly dose information can be incorporated into these parameters. Following analyses that examined the optimal incorporation of smoking dose information into the TSCE model, the following dose–response equation was used [7, 10],

$$\theta_{tobacco} = \left\{ {\theta \times \left( {1 + \theta_{c} \times dose^{{\theta_{p} }} } \right)} \right\}$$

where *dose* is given in cigarettes per day, ${\uptheta }_{tobacco}$ is the parameter of interest with dose information incorporated, $\uptheta$ is the background parameter, ${\uptheta }_{c}$ is a dose–response coefficient effect, and ${\uptheta }_{p}$ is a dose–response power effect.

### Estimation/Fitting Procedure

We used maximum likelihood approaches to fit the model. Specifically, we estimated model parameters that maximized the cohort likelihood, which was constructed as the product of individual likelihoods. Individual likelihoods were calculated as follows,

$$L_{i} \left( {al_{i} ,ae_{i} ;\theta \left( {d_{i} } \right)} \right) = \left\{ {\begin{array}{*{20}c} { - \frac{{S^{\prime}\left( {al_{i} ;\theta \left( {d_{i} } \right)} \right)}}{{S^{\prime}\left( {ae_{i} ;\theta \left( {d_{i} } \right)} \right)}} \text{ for cases,}} \\ { \frac{{S^{\prime}\left( {al_{i} ;\theta \left( {d_{i} } \right)} \right)}}{{S^{\prime}\left( {ae_{i} ;\theta \left( {d_{i} } \right)} \right)}} \text{ for non cases,}} \\ \end{array} } \right.$$

where ${L}_{i}$ is individual likelihood, $a{l}_{i}$ is age of study exit, $a{e}_{i}$ is individual's age of study entry, and $\uptheta \left({d}_{i}\right)$ is the estimated initiation, promotion, and malignant conversion rates for that individual with their specific dose history [7]. For more details on survival (S) and likelihood calculations, see Supplementary Methods.

Then, using MEC data, the total likelihood calculated as,

L = $${\prod }_{i}{L}_{i}\left(a{l}_{i},a{e}_{i};\uptheta \left({d}_{i}\right)\right)$$

was maximized.

We used Davidson-Fletcher-Powell algorithms in the Bhat package in R to optimize the likelihood. We fitted multiple models, allowing for differences in race/ethnicity and gender for all parameters, background malignant conversion and promotion only, and background and coefficient tobacco effects for malignant conversion and promotion. We also fitted models allowing for differences solely by race/ethnicity or gender. We used Likelihood Ratio tests (for nested models) and the Akaike Information Criterion (AIC) (for unnested models) to select the best fitting models.

### Sensitivity Analysis

For the main analysis, we censored individuals after 10 years of their last smoking assessment. To assess the impact of this assumption, we conducted a sensitivity analysis whereby individuals were censored 5, 15, and 30 years after last complete questionnaire.

## Results

As determined by likelihood ratio tests and the AIC, the best model was one in which background parameters, the promotion smoking coefficient, and the malignant conversion smoking coefficient differed by both race/ethnicity and gender. Smoking effects on tumor initiation did not significantly improve model fit; thus, the final model only includes smoking-related promotion and malignant conversion, consistent with previous analyses of lung cancer incidence in other contemporary prospective cohorts [7, 11]. Due to the uncertainty of the estimates of the dose–response power for malignant conversion, this parameter was fixed to the dose–response power for malignant conversion obtained in a previous TSCE model analysis of two large U.S. cohorts [7]. Additionally, the best model selected was one in which the promotion smoking dose–response power did not differ by race/ethnicity and gender.

**Fig. 2:**
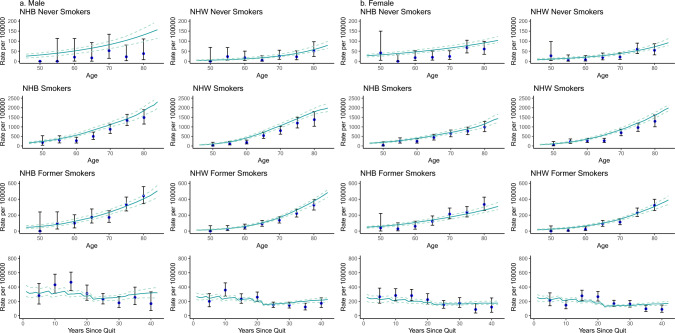
Lung cancer incidence of NHB and NHW individuals in the Multi-Ethnic Cohort (MEC). Solid line represents the model prediction. Dashed lines are MCMC 95% CI. Blue dots are the observed MEC incidence summed over five-year interval with 95% CI calculated based on Poisson assumptions

Table 2 shows parameter estimates and 95% confidence intervals which were estimated with MC MC simulations utilizing the Metropolis Hasting algorithm [7, 28]. Figure 2 shows that the model replicates lung cancer incidence well for NHB and NHW males and females by smoking status.

**Table 2:** Table of TSCE parameter estimates. Estimate (*95% Confidence Intervals*). See supplement for precise definitions and of${\upmu }_{0}$,${\upmu }_{1}, \text{g},$and$\upbeta$

Table of TSCE parameter estimates for Non-Hispanic Black vs. Non-Hispanic White					
	Parameter	Non-Hispanic Black		Non-Hispanic White	
		Male	Female	Male	Female
Background rate	Initiation and malignant conversion rate ${\upmu }_{0}={\upmu }_{1}$	4.058E-07 (2.502E-07, 6.576E-07)	5.282E-07 (*3.845E-07,* *7.253E-07*)	8.836E-08 (*4.723E-08, * *1.652E-07*)	1.758E-07 (*1.057E-07,* *2.922E-07*)
	Initiated cells promotion rate g= ∝−β−μ_0_	0.0454 (*0.0312, 0.0656*)	0.0299 (0.0194, 0.0458)	0.0803 (*0.0639, 0.1004*)	0.0626 (*0.0483, 0.0809*)
Fixed parameters	Stem cell population X	10^7			
	Initiated cells’ division rate ∝	3			
Tobacco coefficients	Tobacco promotion rate coefficient g_c_	0.3306 (*0.1995,* *0.4946*)	0.5646 (*0.2708,* *0.8190*)	0.2623 (*0.2168,* *0.3136*)	0.2837 (*0.2148,* *0.3644*)
	Tobacco promotion rate power g_p_	0.4013			
	Tobacco malignant conversion rate coefficient ${\upmu }_{1c}$	0.4160 (*0.2636,* *0.6507*)	0.2871 (*0.1672,* *0.4889*)	0.2691 (*0.1661,* *0.4334*)	0.2565 (*0.1563,* *0.4182*)
	Tobacco malignant conversion rate power ${\upmu }_{1p}$	0.4684*			
Loglik			16,775.3		

*denotes Meza et al.[7] analysis estimates fixed in this study

### Model Parameter Estimates

The best fitted model allowed for differences in background parameters by race/ethnicity and gender, with the differences being more substantial by race/ethnicity than by gender. NHB males have significantly higher background initiation and malignant conversion rates than NHW males, with 4.058E-07 (95% CI: 2.502E-07,6.576E-07) vs. 8.836E-08 (95% CI: 4.723E-08, 1.652E-07), respectively. Background promotion was slightly higher for NHW males compared to NHB males. Similar patterns were observed for females. No major differences were observed when comparing males and females within each racial group. The estimated promotion rates were somewhat higher in males compared with females in both NHB and NHW individuals; however, the differences were not statistically significant. In contrast, the initiation & malignant conversion rates were higher in females than in males in both NHB and NHW, with the gender difference being statistically significant only in NHW individuals.

The best fitted model allowed for differences in tobacco effects on promotion and malignant conversion by race/ethnicity and gender. Differences between males and females for both tobacco promotion rate coefficient and tobacco malignant conversion rate were not substantial. However, NHB males had a higher tobacco promotion rate coefficient compared to NHW males, with 0.3306 (95% CI: 0.1995, 0.4946) vs. 0.2623(95% CI: 0.2168,0.3136) respectively. Additionally, NHB males had a higher tobacco malignant conversion rate coefficient compared to NHW males, with 0.4160 (95% CI: 0.2636, 0.6507) vs. 0.2691(95% CI: 0.1661, 0.4334), respectively. Similarly, tobacco promotion rate and malignant conversion rate coefficients were higher for NHB females compared to NHW females.lkn

### Never Smokers

**Fig. 3:**
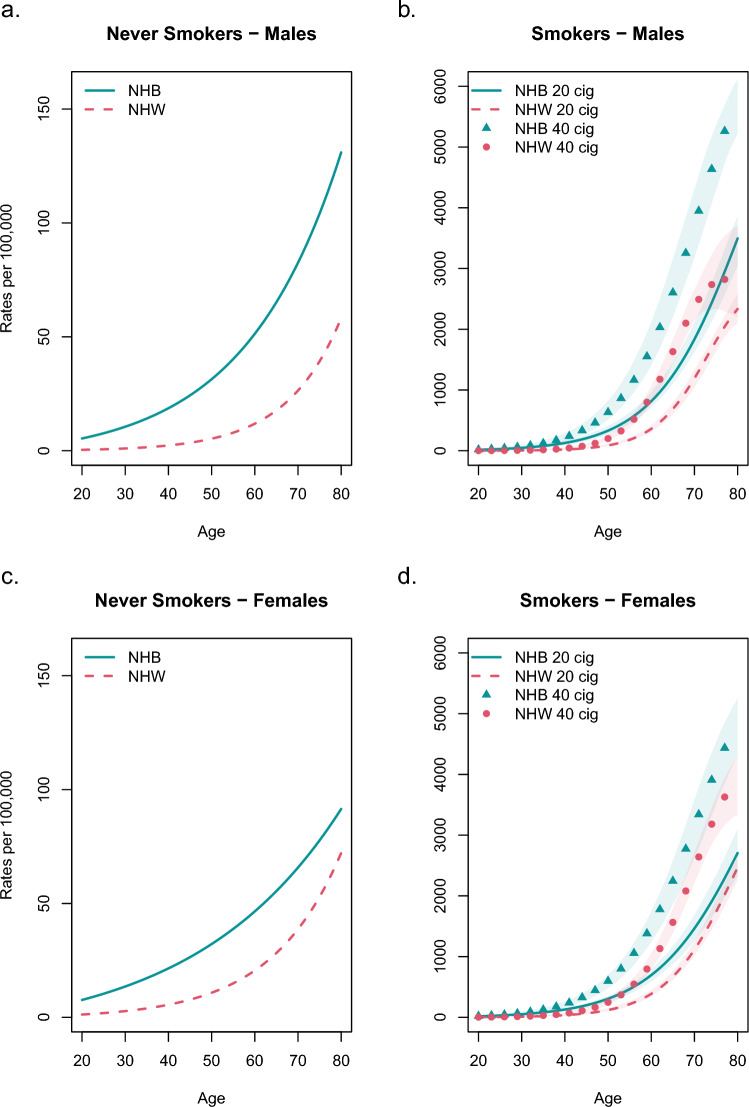
**A** and **C** show simulated incidence curves for never smokers. **B** and **D** show simulated incidence for an individual who smokes 20 cigarettes starting at age 20 and 40 cigarettes starting at age 20. Shaded regions are MCMC 95% CI

The top panels of Fig. 2a, b show lung cancer incidence for never smokers in MEC data. Figure 3a, c show the model prediction of lung cancer incidence for never smokers. NHB never smokers had a higher risk of lung cancer compared with NHW never smokers. This is attributable to the higher background initiation and malignant conversion rates for NHB individuals.

### Smokers

The second panels of Fig. 2a, b show lung cancer incidence for smokers in MEC data. Figure 3b, d show the model prediction of lung cancer incidence for individuals who smoke 20 and 40 cigarettes daily starting at age 20. At every age, NHB smokers had a higher incidence of lung cancer than NHW smokers for both smoking histories. As expected, there was a dose–response: those who smoked 40 cigarettes per day have a higher incidence than those who smoked 20 cigarettes per day.

**Fig. 4:**
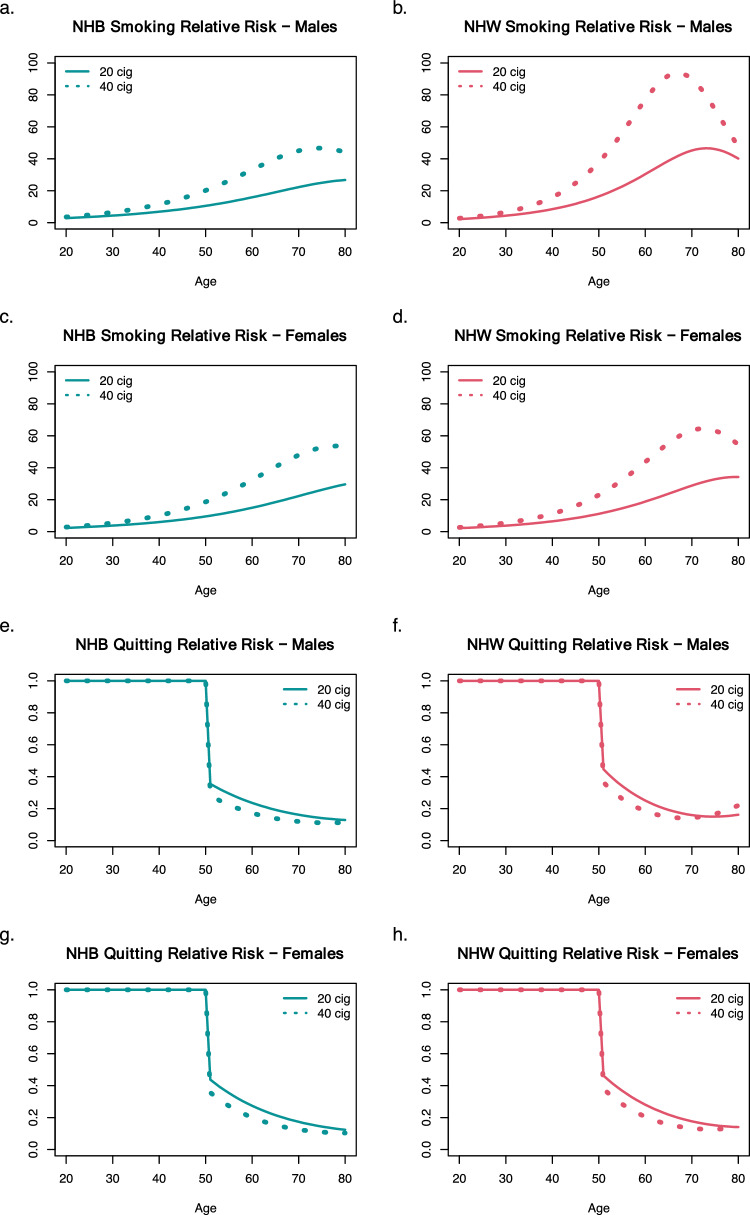
**A**, **B**, **C**, and **D** show the relative risk of lung cancer between smokers and never smokers for NHB males, NHW males, NHB females, and NHW females, respectively. **E**, **F**, **G**, and **H** show the relative risk of lung cancer between smokers and individuals who quit at age 50

Figure 4a, b, c, d show the relative risk (RR) of lung cancer between smokers and never smokers by gender and race/ethnicity. As expected, duration impacted the risk of lung cancer: the RR of lung cancer comparing smokers to never smokers increased as smoking years accumulated. The later decline can be attributed to a faster increase in never smoker lung cancer risk with age in older ages, relative to smokers. NHB individuals had lower RR due to duration compared with NHW individuals which may reflect the strong influence of background risk among NHB individuals.

Supplementary Figures S1a, b, c, and d show the hazard ratio of NHB smokers compared to NHW smokers. For NHB males compared to NHW males, the hazard ratio and MCMC confidence intervals were above 1 for all ages. The hazard ratio was marginally smaller between those who smoked 20 cigarettes per day and those who smoked 40 cigarettes per day.

### Former Smokers

Figure 4e, f, g, h show the RR of lung cancer between a simulated continuing smoker and an individual who quit smoking at age 50. Tobacco effects on malignant conversion immediately impacted lung cancer incidence; thus, quitting had a strong and immediate effect on incidence. Many years after quitting, the effects of promotion were reflected by the continuing decrease in RR of former vs current smoker. There was little difference in this effect by race/ethnicity and gender. However, the effect of tobacco on malignant conversion was highest for NHB males (Table 2), which was reflected in a slightly greater decrease in relative risk after quitting.

Supplementary Figure S2 shows the relative risk (RR) of lung cancer between former smokers and never smokers by gender and race/ethnicity. While relative risk declines for individuals who quit smoking, risk remains high compared to never smokers (relative risk > 1), even many years after quitting. NHW current smoking males have substantially higher relative risk compared to NHB current smoking males, likely due to the higher risk among NHB never smokers. There was little difference between NHB and NHW females.

We saw similar patterns in the hazard ratio of NHB compared to NHW former smokers (Supplementary Figure S3) as we did for smokers (Supplementary Figure S1). The hazard ratio for NHB males compared to NHW males is above 1 for all ages with MCMC confidence intervals at or above 1. At younger ages, the hazard ratio was greater and then decreased as age increased. The hazard ratio was slightly reduced at 40 cigarettes per day compared with 20 cigarettes per day. Similarly, for females, the hazard ratio decreased with age. However, the hazard ratios were generally smaller for females compared to males.

### Sensitivity Analysis

Results were consistent: findings for smoking-related and background patterns with 10-year censoring were maintained for 5, 15, and 30-year censoring (data not shown).

## Discussion

This analysis showed that lung cancer incidence differs between NHB and NHW individuals for both smokers and never smokers. Generally, NHB individuals had slightly higher smoking-related promotion and non-smoking-related (background) initiation effects than NHW individuals suggesting that background risk not dependent on smoking plays a significant and under-recognized role in explaining racial differences. Because background initiation and malignant conversion effects were higher in the NHB population, further research into disparities needs to consider non-smoking-related risk factors: i.e., environmental, occupational, and other carcinogenic exposures [29, 30]. Current research recognizes that Black individuals are exposed to more particulate air matter[29] and more likely to live near sources of pollution such as high traffic roads [29–34]. Factors of systemic racism, such as redlining, which is defined as the denial of mortgage loans based on locations with majority Black populations [35–37], may play into this higher burden of exposure in Black Americans [37, 38]. Furthermore, a recent analysis found that airborne particulate matter affected lung cancer risk in never smokers by acting on promotion [39]. Our results showed that more research is needed to, first, identify racial differences in non-smoking exposures related to lung cancer risk, second, investigate how these exposures act on the pathways of carcinogenesis, and third, understand how they interact with smoking-related mechanisms.

### Study Strengths

No previous analyses of lung cancer incidence using the TSCE model have compared age and time-dependent risk patterns between NHB and NHW populations. The TSCE model allowed for consideration of detailed individual smoking history, including changes in dose over the life course. Additionally, the TSCE model captured the potential impact of smoking and non-smoking factors on the cancer development process for NHB & NHW populations.

### Study Limitations

The MEC data are limited to individuals from California and Hawaii and thus may not be representative of the overall US NHB and NHW populations. However, the relationships estimated between smoking and lung cancer risk likely apply consistently across individuals with similar racial/ethnic backgrounds. Additionally, other tobacco products, such as cigars, were not evaluated in this analysis and may impact cigarette use as well as lung cancer risk. Additionally, to incorporate dose into our model, we converted a categorical smoking variable to be continuous which may increase noise in our estimates. Finally, certain smoking behaviors could not be considered in the models: inhalation depth, puffs per minute, and menthol use. Of note, NHB individuals are much more likely to smoke menthol cigarettes than their NHW counterparts [5, 40] . However, menthol cigarettes have not been shown to increase lung cancer risk compared to regular cigarettes [41]. These factors might play a role in shaping the observed and estimated differences in risk between NHB and NHW populations. Furthermore, genetics and familial risk, which have not been accounted for in this analysis, have been shown to play a role in lung adenocarcinoma and likely play a role in squamous cell carcinoma [42, 43]. However, there is insufficient evidence to support these factors playing a role in racial differences [44]. Here, it is worth noting that race, considered a social construct devoid of biological basis, is a proxy for the impact of structural and individual experiences of racism [44].

### Relationship to Other Literature

#### Never Smokers

Few studies have examined lung cancer incidence among never smokers by race/ethnicity due to data or sample size limitations; there are few data sources that collect information on race/ethnicity, smoking history, and lung cancer outcomes and have sufficiently large samples of NHB individuals to obtain stable estimates. Previous MEC analyses have found no difference in risk by race/ethnicity or gender among never smokers [3, 45]. Of the existing studies, Pinheiro et al. found slightly higher risk among black never smokers than white never smokers [46], while Thun et al. found that black females had higher incidence than white females but drew no conclusions on males due to small samples [47]. Our findings add to this growing body of literature and suggest that black never smokers have higher risk compared to white never smokers. Furthermore, there is growing evidence of the relationship between air pollution and lung cancer among never smokers [48, 49]. Thus, research and interventions should be focused on understanding and reducing disparities in air pollution exposure and other environmental exposures.

#### Smokers

As expected, both dose and duration impacted the risk of lung cancer. Those who smoked more had higher incidence than those who smoked less, which aligns with previous MEC analyses [3]. However, when comparing NHB and NHW individuals, we observed a higher hazard ratio for those who smoked 20 cigarettes per day compared to those who smoked 40 cigarettes per day. This finding also aligns with previous MEC analyses which showed reduced differential hazards with a greater smoking dose [3]. In this analysis, similar patterns were observed for females, although the hazard ratio was generally slightly lower for females than males [3].

As in previous studies, the initial increase in RR with duration of smoking can be directly attributed to the strong influence of tobacco on promotion [7, 50]. Additionally, NHB individuals had lower RR due to duration compared with NHW individuals which may reflect the strong influence of background risk among NHB individuals.

#### Former Smokers

Quitting smoking resulted in a rapid decrease in lung cancer risk relative to continuing smoking (Fig. 4) which is consistent with a previous analysis of US cohorts using the TSCE [7, 11]. In particular, Hazelton et al. found similar decreases in lung cancer risks after quitting in the British Doctors cohort and the CPS-I and CPS-II cohorts, consistent with the empirical data. Similarly, Meza et al. found rapid decreases in lung cancer risk in former versus former smokers after quitting in the Nurses’ Health Study and the Health Professionals’ Follow-up study. Risk continues to drop with increasing years since quit when comparing former smokers to current smokers (Fig. 4). In contrast, other analyses of the MEC study have found slower decreases after quitting in the excess relative risk (ERR) of lung cancer, with an estimated 5–6% decrease in current vs former smokers ERR [3]. We attribute this to the use of different methodologies and the potential impact of reverse causality, with quitting occurring due to the onset of lung cancer symptoms [51], a feature not allowed by the constraints in the TSCE model, where smoking promotion and malignant conversion decrease immediately after quitting. Regardless, a decrease in ERR of 6% after 30 years would result in a RR between former vs current smokers of 17%, consistent with the long-term findings of the TSCE model presented here as well as others. It is important to highlight that while risk decreases for those who quit versus those who continue smoking, former smoker risk remains high compared to that of never smokers (Supplementary Fig. 2). This aligns with results from previous analyses that show high risk for former smokers even years after quitting [52, 53]. If risk remains high for many years after quitting, current lung cancer screening criteria which only includes those who have quit in the past 15 years may not be optimal for preventing lung cancer mortality. Recently, in recognition of the high risk for former smokers beyond the 15 year quit threshold, the American Cancer Society removed years since quit screening criteria for lung cancer screening guidance [54, 55].

### Applications

The model presented here provides much-needed information that can be used to evaluate primary, secondary, and tertiary lung cancer interventions. Like previous analysis of gender differences in lung cancer incidence in the US using the TSCE model [7], this analysis was conducted within the Cancer Intervention and Surveillance Modeling Network (CISNET) Lung Working Group. CISNET is an NCI-sponsored consortium of simulation modelers who investigate the effect of preventive interventions on cancer risk. The current race/ethnicity-based incidence model is a valuable extension of risk models incorporated into CISNET lung cancer natural history simulation models, as previous TSCE models have been [56].

Furthermore, the previous TSCE model was essential when the CISNET Lung Working Group conducted decision analyses, which provided supporting evidence in updating the United States Preventive Services Task Force (USPSTF) lung cancer screening recommendations [57]. USPSTF A & B recommendations need to be covered by private insurers [58]. These screening recommendations were developed for the general US population, but optimal strategies may differ by race/ethnicity. New USPSTF screening guidelines published in 2021 increased the proportion of eligible NHB individuals screened by extending the screening criteria to include younger ages 50–54 and less heavy smokers with 20–29 pack-years of smoking history compared to the previous 2013 screening guidelines; however, racial disparities in screen-eligibility may still remain [59–61]. Thus, there is a need for models specific to the NHB population which will help evaluate the effect of screening for this sub-population more accurately [35, 62, 63]. The incidence model presented in this paper is, thus, a significant contribution to developing an NHB-specific lung cancer natural history model, which will be used to examine different screening strategies in the NHB population.

In summary, this analysis provides insights into the biological and epidemiological mechanisms underlying lung cancer incidence differences between NHB and NHW individuals. The analysis suggests that while smoking plays an important role in lung cancer risk, background risk not dependent on smoking also plays a significant and under-recognized role in explaining racial differences. Ultimately, the resulting TSCE model will be used to inform race/ethnicity-specific lung cancer natural history models to assess the impact of preventive interventions on US lung cancer outcomes and their disparities by race/ethnicity.

## Supplementary Information

Below is the link to the electronic supplementary material.

Supplementary file1 (DOCX 140 KB)

## Data Availability

The datasets analyzed during the current study may be accessed through application for MEC data, please see: https://www.uhcancercenter.org/for-researchers/mec-data-sharing. R code to compute TSCE hazard and survival are available from the authors by request.
