# Supplementary material for: Analysis of Lung Cancer Incidence in Non-Hispanic Black and White Americans using a Multistage Carcinogenesis Model
Source: Cancer Causes Control. 2024 Nov 19;36(3):285–96. doi: 10.1007/s10552-024-01936-7 (PMC11928365; doi:10.1007/s10552-024-01936-7)
Supplement: Supplementary file 1 — Supplementary file1 (DOCX 140 KB) [file 10552_2024_1936_MOESM1_ESM.docx]

**SUPPLEMENTARY MATERIAL**

**Table S1:** MEC Study participants completing Follow-up Questionnaires.

| Number of Questionnaires Completed | Non-Hispanic Black | | Non-Hispanic White | |
| --- | --- | --- | --- | --- |
|  | Number who died or were diagnosed with lung cancer (percent of individuals completing questionnaires) | Number of participants (percent of total participants) | Number who died or were diagnosed with lung cancer (percent of individuals completing questionnaires) | Number of participants (percent of total participants) |
| 1 (Baseline only) | 1066 (5.7%) | 18694 (56.5%) | 1003 (5.5%) | 18359 (37.8%) |
| 2 | 229 (3.1%) | 7507 (22.7%) | 356 (3.4%) | 10440 (21.5%) |
| 3 | 99 (1.4%) | 6866 (20.8%) | 237 (1.2%) | 19720 (40.6%) |

Note for column “Number who died or were diagnosed with lung cancer (percent of individuals completing questionnaire)”: The percent is calculated as the number of people who died or were diagnosed with lung cancer over the number of participants who answered 1, 2 or 3 questionnaires depending on the row.

**Fig. S1** Hazard ratio for NHB vs. NHW smokers. This figure displays relative hazards comparing a simulated risk for a NHB smoker over a NHW smoker. Figures 1A and 1B are male smokers and Figures 1C and 1D are female smokers. Dashed lines are MCMC 95% CI. A grey horizontal line shows RR of 1.

**Fig. S2** Figures 2A, 2B, 2C, and 2D show the relative risk of lung cancer between former smokers (quitting at age 50) and never smokers for NHB males, NHW males, NHB females, and NHW females, respectively. A grey horizontal line shows RR of 1.

**Figure S3:** Hazard ratio for NHB vs. NHW former smokers. This figure displays relative hazards comparing a simulated risk for a NHB former smoker over a NHW former smoker. Figures 3A and 3B are male former smokers and Figures 3C and 3D are female former smokers. Dashed lines are MCMC 95% CI. A grey horizontal line shows RR of 1.

**Supplementary Methods**

**MEC**

In Table 1 readers may notice that the maximum age at study entry is beyond the recruitment ages designated by the Multiethnic Cohort (MEC) study. The MEC recruited individuals aged 45 to 80. In our analysis, for individuals that did not complete baseline questionnaire but did complete questionnaire 3, we included them into our analysis at the age of their questionnaire 3 and thus these individuals may have age at entry higher than age 80.

**TSCE Model**

The TSCE model captures the carcinogenic process of initiation, progression, and malignant conversion. Cells become initiated according to a Poisson process where intensity ($v)$ for this process is given by:

$v\left( t \right)=X\left( t \right)\times\mu_{0}$(t)

where *X* is the number of healthy cells and $\mu_{0}$ is the initiation rate per cell at a specific time.^59^

Once cells have entered the initiated or pre-cancerous phase, they can reproduce with rate $\alpha$ or die with rate $\beta$. Finally, they become malignant with rate $\mu_{1}$. These parameters are not identifiable on their own but can be packaged into three identifiable parameters labeled *p*, *q*, and *r*^59^:

$$p,q=\frac{1}{2}\left( -\left( \alpha-\beta-\mu_{1} \right)\mp\sqrt{\left( \alpha-\beta-\mu_{1} \right)^{2}+4\alpha\mu_{1}} \right)$$

$$r=\frac{\mu_{0}X\left( 0 \right)}{\alpha}$$

In this case, the hazard and survival for the TSCE model can be calculated explicitly as

$$S\left( t \right)=\exp\left\{ \sum_{j=1}^{k} \frac{\mu_{0}X}{\alpha}ln\left( \frac{q_{j}-p_{j}}{f_{j}\left( t_{j-1},t_{k} \right)} \right) \right\},$$

where *f_j_* represents a recursive function derived to identify time-dependent parameters and further explained in Meza et al. and Heidenreich et al.^7,59^

Although the parameters of interest are not identifiable on their own, the parameters *p, q*, and *r* can be interpreted as representing the effects of initiation, promotion, and progression. For example, the parameter $\alpha$is set to 3 for identifiability issues in this model. If $\alpha$ is fixed and $X\left( 0 \right)$, the number of healthy cells at time 0, is fixed then *r* is proportional to $\mu_{0}$. The promotion rate can be described by $\alpha-\beta$, and p approximates –( $\alpha-\beta$ ), so *p* is proportional to promotion.^60,61^ The parameter *q* can be approximated as $\mu_{1}/$(1 - $\frac{\beta}{\alpha}$), so *q* is proportional to $\mu_{1}$.

The parameters *p, q*, and *r* can be estimated by fitting the model to prospective cohort data using the TSCE hazard and survival formulas described in Heidenreich et al. and Meza et al. ^7,59^. These formulas can account for piecewise constant age-varying parameters. This allows for modeling of the impact of age-dependent exposures, such as changing smoking behaviors, which might affect the background rates of initiation, promotion, and malignant conversion. Additional identifiability issues are introduced with time-dependent factors, so we must set constraints on the parameters.^59^ A common way to handle this identifiability issue is to set the initiation rate ($\mu_{0}$) equal to the malignant conversion rate ($\mu_{1}$), which was done in a previous analysis of lung cancer incidence by Meza et al.^7^ To obtain an identifiable set of parameters, we set the initial healthy cell population to 10^^7^, initiated cell division or $\alpha$ to be 3, and then initiation and malignant conversion to be equal to each other ($\mu_{0}, =\mu_{1})$ as in Meza et al.^7^

**Supplementary Chart 1**: Flow Chart documenting inclusion and exclusion criteria and individuals in the final sample of NHB and NHW individuals.
